# Supplementary material for: Chromosome conformation capture resolved near complete genome assembly of broomcorn millet
Source: Nat Commun. 2019 Jan 25;10:464. doi: 10.1038/s41467-018-07876-6 (PMC6347627; doi:10.1038/s41467-018-07876-6)
Supplement: Supplementary file 1 — Supplementary Information [file 41467_2018_7876_MOESM1_ESM.pdf]

Chromosome conformation capture resolved near complete genome assembly of broomcorn millet

Shi et al.

Supplementary Note 1

K-mer estimation of genome size

To estimate the genome size of Longmi4, we generated oversaturated Illumina pair end reads (150 bp, ~116 x), which could be used for both k-mer analysis and genome polish. Firstly, we filtered the low quality reads and bases by SolexaQA[1] (v.2.5), then count the k-mer depth using jellyfish[2] (v2.2.6) with parameters -m 17 -s 200M -C. We plotted the k-mer depth against the k-mer count (**Supplementary Figure 2**), and found the genome was highly homozygous since no peak was detected around half the depth of the major peak. The heterozygosity ratio of Longmi4 genome was estimated to be ~0.04% by GenomeScope[4] (http://qb.cshl.edu/genomescope/). A potential tetraploid genome was further inferred, since a secondary peak was detected around two times the depth of the major peak. We calculated the k-mer coverage to be 74,578,127,218, and the average k-mer depth to be ~84x since it theoretically follows the Poisson's distribution. Finally, the genome size was estimated to be ~887.8 Mb according to the formula that Genome_Size = K-mer coverage/Average k-mer depth[3].

Supplementary Note 2

Genome assembly of Longmi4 by PacBio reads and BioNano optimal maps

We used Falcon[5] (v1.8.7) to assemble the raw Pacbio reads into contigs with 4 main steps: a) Raw reads overlapping for error correction; b) Pre-assembly and error correction; c) Overlap detection and filtering; d) Graph construction and contigs generation. In order to optimize the assembly results, we tried a variety of parameters and finally assembled the raw contigs with the following paramerters: length_cutoff =11 Kb, length_cutoff_pr =15 Kb, pa_HPCdaligner_option = -v -B128 -M24 -t12 -e.75 –k18 -w8 –h180 -T32 –l2800 -s1000, ovlp_HPCdaligner_option = -v -B128 -t12 -h280 -e.96 -k22 -T32 -l3200 -s1000. The total length of raw contigs was ~838 Mb, including 1,262 contigs with N50 ~2.58 Mb. The statistics of raw contigs were listed in **Supplementary Table 11**.

The raw contigs contained a variety of sequencing errors, with an estimated identity of ~97% as compared with the final high-quality consensus contigs. So, the original PacBio reads were mapped back to the raw contigs with Blasr[6] (v5.1), a mapper with high tolerance of sequencing errors, with the following parameters (--bam --bestn 5 --minMatch 18 --nproc 4 --minSubreadLength 1000 --minAlnLength 500 --minPctSimilarity 70 --minPctAccuracy 70 --hitPolicy randombest --randomSeed 1). Then, the raw contigs were corrected by Arrow (v2.1.0) with the parameter -j 30 (https://github.com/PacificBiosciences/GenomicConsensus). After the first round polish with PacBio reads, the identity of contigs was estimated to be higher than ~98%, so we mapped the Illumina reads back to the contigs with bwa mem (v0.7.12)[7] by default parameters, then corrected with Pilon[8] (v1.20) to generate the final consensus contigs (--genome reference.fasta --changes --vcf --diploid --fix bases --threads 40 --mindepth 20).

To further anchor the contigs into scaffolds, we generated the BioNano optimal maps with a data volume of ~208.8 Gb (N50 ~255.2 Kb, **Supplementary Table 1 and Supplementary Table 2**). We firstly mapped the clean BioNano data back to the consensus contigs by IrysSolve (BioNano Genomics), with a mapping rate of ~17.5%. The BioNano data were further assembled into optimal physical maps with a Consensus Genome Map length of ~864.3 Mb and N50 ~1.45 Mb. After aligned the assembled optical genome map back to the contigs and resolved the conflicts, we generated the final assembly with 905 scaffolds and 1,308 contigs. The detailed assembly statistics of conflicts-resolved contigs and scaffolds were listed in **Supplementary Table 12**.

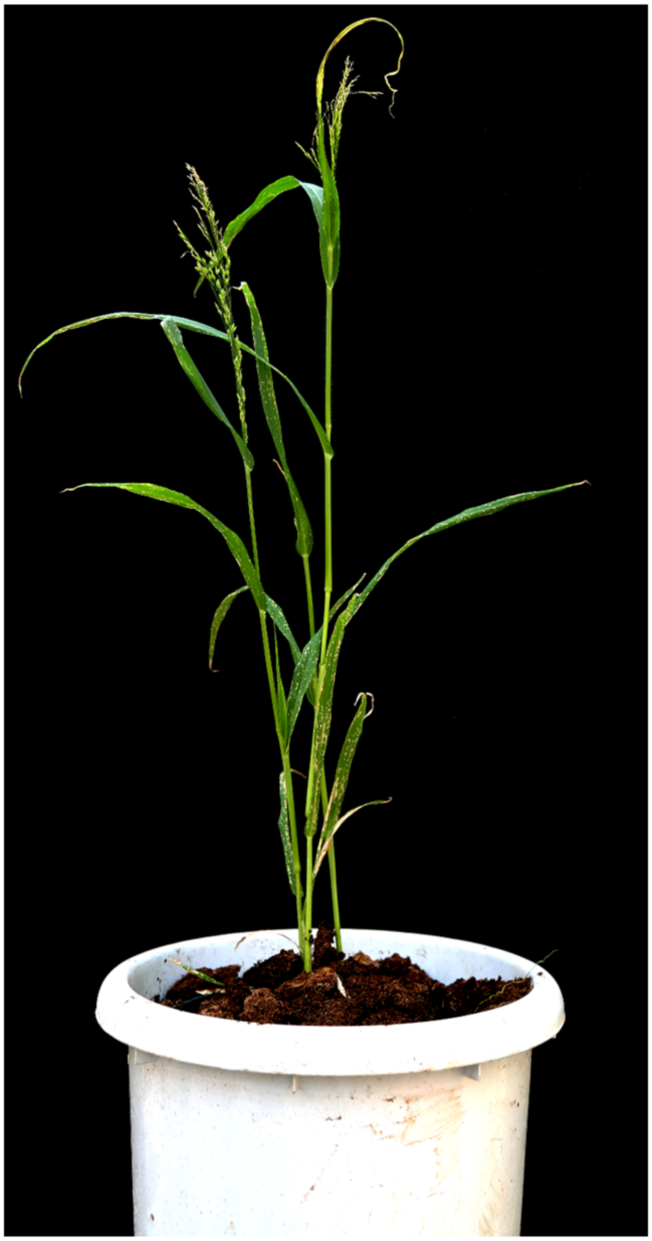

Supplementary Figure 1. The phenotype of Longmi4 at the flowering stages (52 days after sowing).

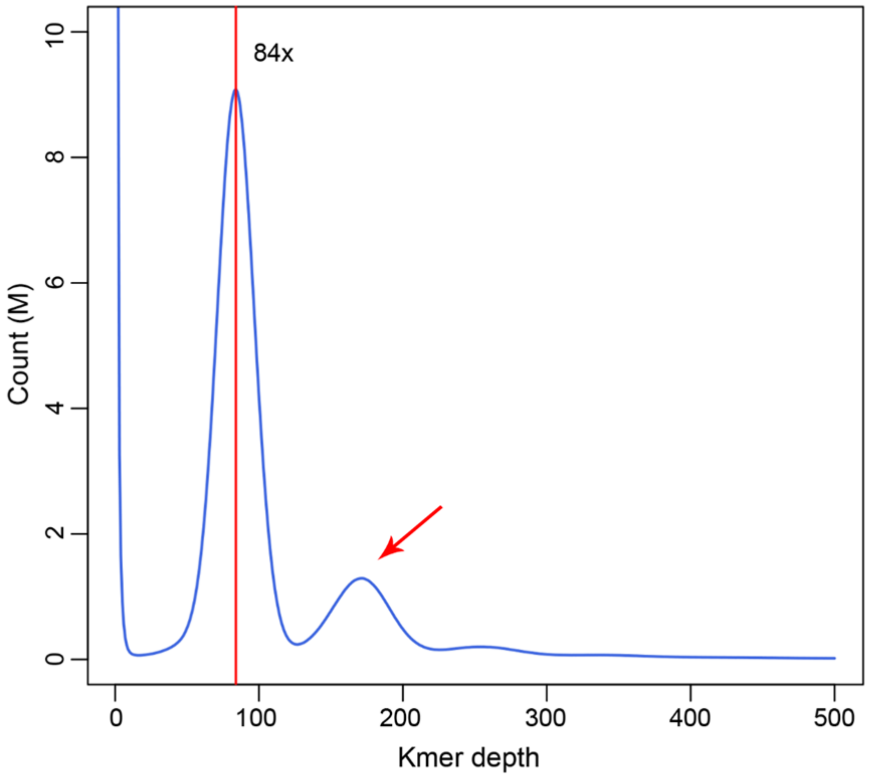

Supplementary Figure 2. K-mer distribution (17-mer) of Illumina reads. Highly homozygous genome (the heterozygosity ratio was ~0.04%) and a potential tetraploid genome (red arrow) were detected from k-mer distribution. The genome size was estimated to be ~887.8 Mb.

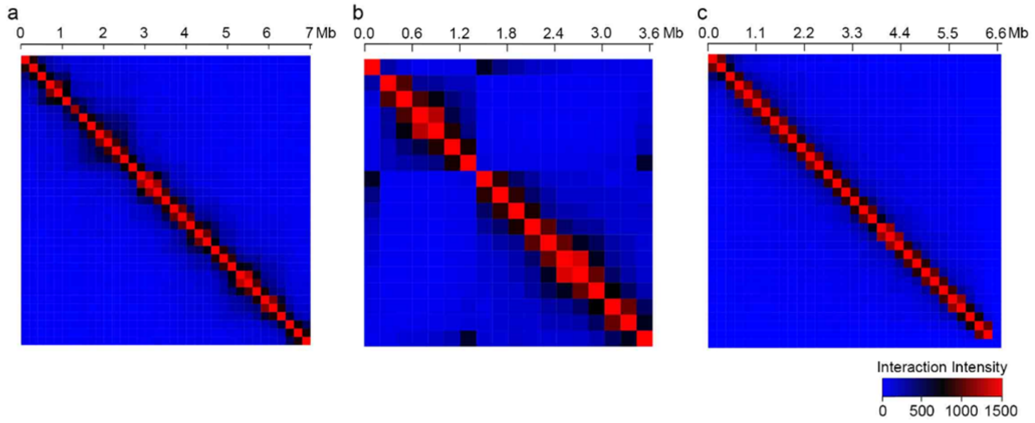

Supplementary Figure 3. The Hi-C interaction matrices within 3 intact scaffolds (resolution = 200 kb). (a) Scaffold_30 (~6.76 Mb). (b) Scaffold_128 (~3.35 Mb) and (c) Scaffold_160 (~6.38 Mb).

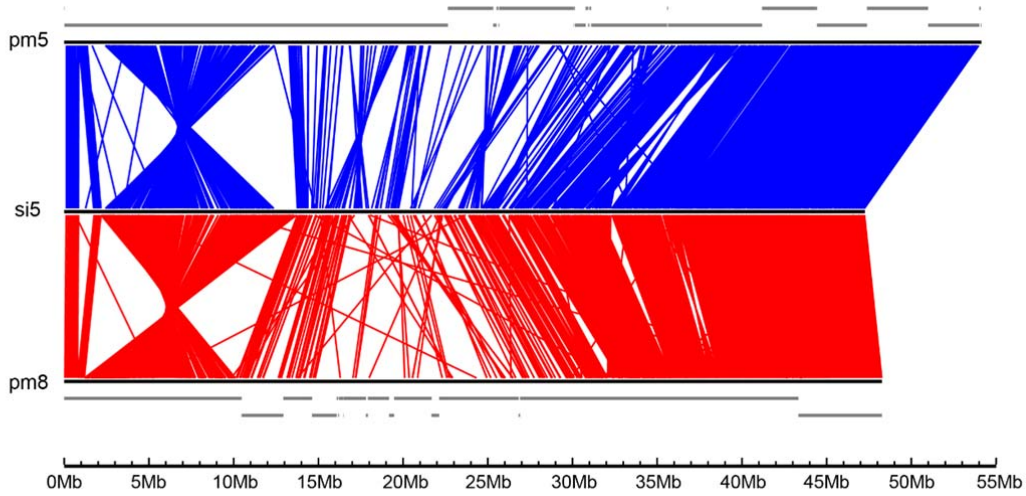

Supplementary Figure 4. Two inversions (~11.8 Mb and ~8.9 Mb) identified on two homologous pseudomolecules (Pm5 and Pm8) that were supported by intact scaffolds. The grey bars denoted the scaffolds anchored onto Pm5 and Pm8.

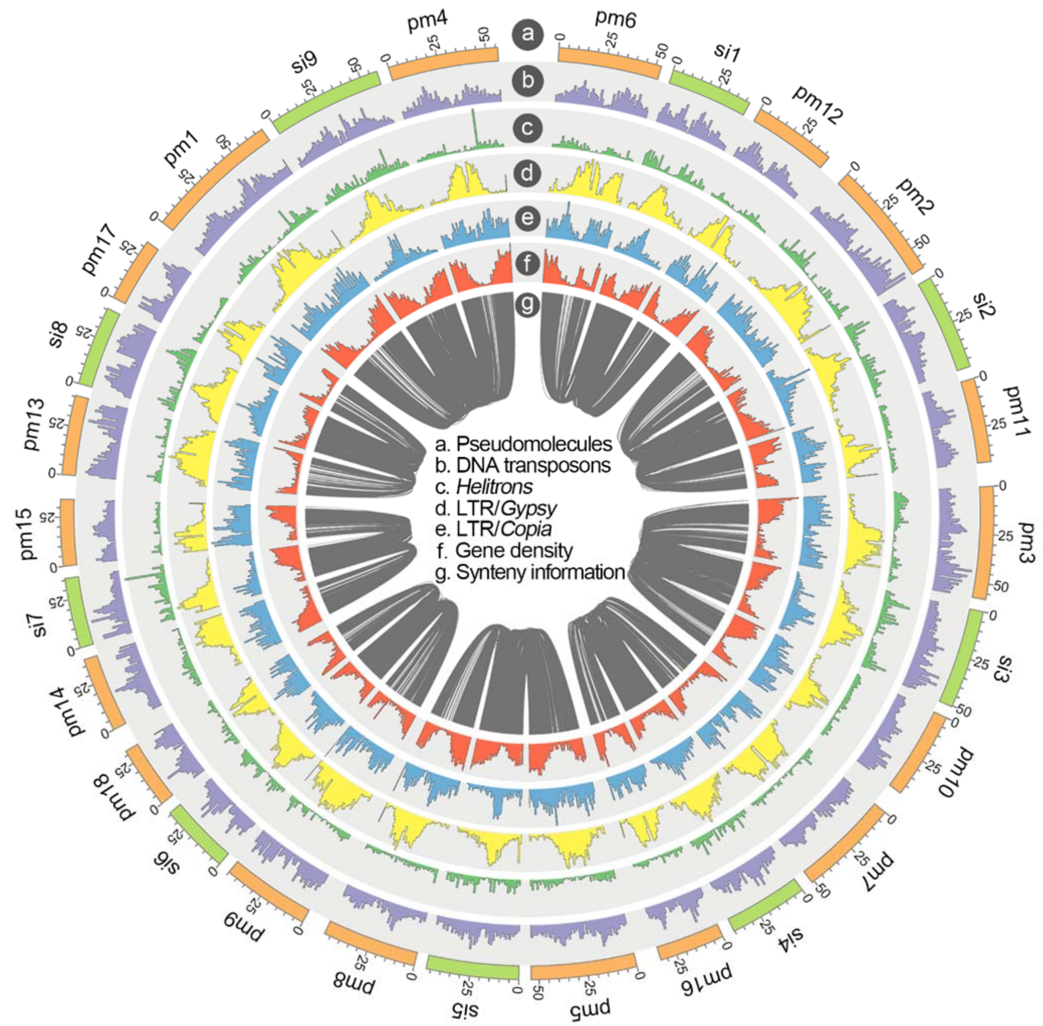

Supplementary Figure 5. The landscape of genome assembly and annotation of broomcorn millet. Tracks from outside to the inner corresponded to: a. Pseudomolecules; b. DNA transposons; c. *Helitrons*; d. *Gypsy*; e. *Copia*; f. Genes and g. Synteny information between broomcorn millet and foxtail millet. Pm, *P. miliaceum*; Si, *S. italica*.

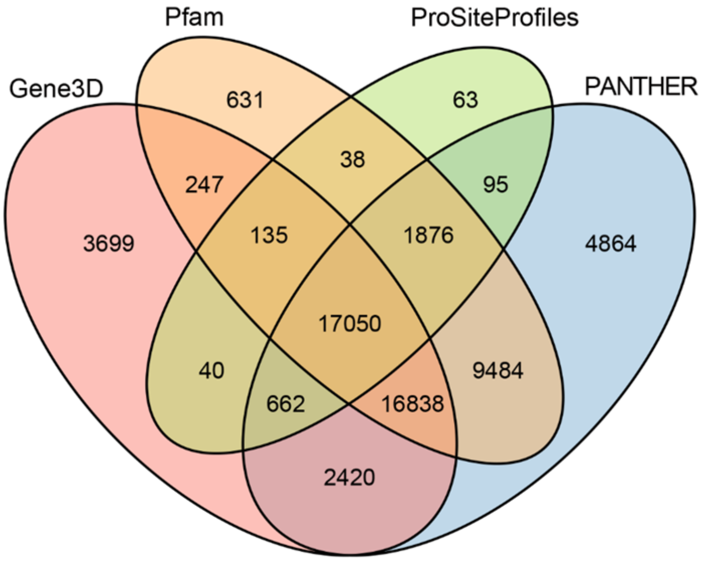

Supplementary Figure 6. The functional annotations of gene models by InterProScan. Gene3D (N = 41,091), Pfam (N = 46,299), ProSite (N = 19,959) and PANTHER (N = 53,289) referred to 4 different sources of annotations from InterProScan.

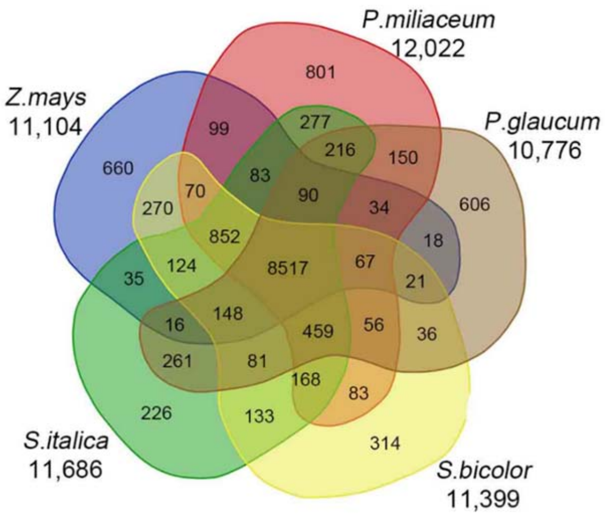

Supplementary Figure 7. The number of shared and specific gene families in broomcorn millet (*P. miliaceum*), foxtail millet (*S. italica*), pearl millet (*P. glaucum*), maize (*Z. mays*) and sorghum (*S. bicolor*).

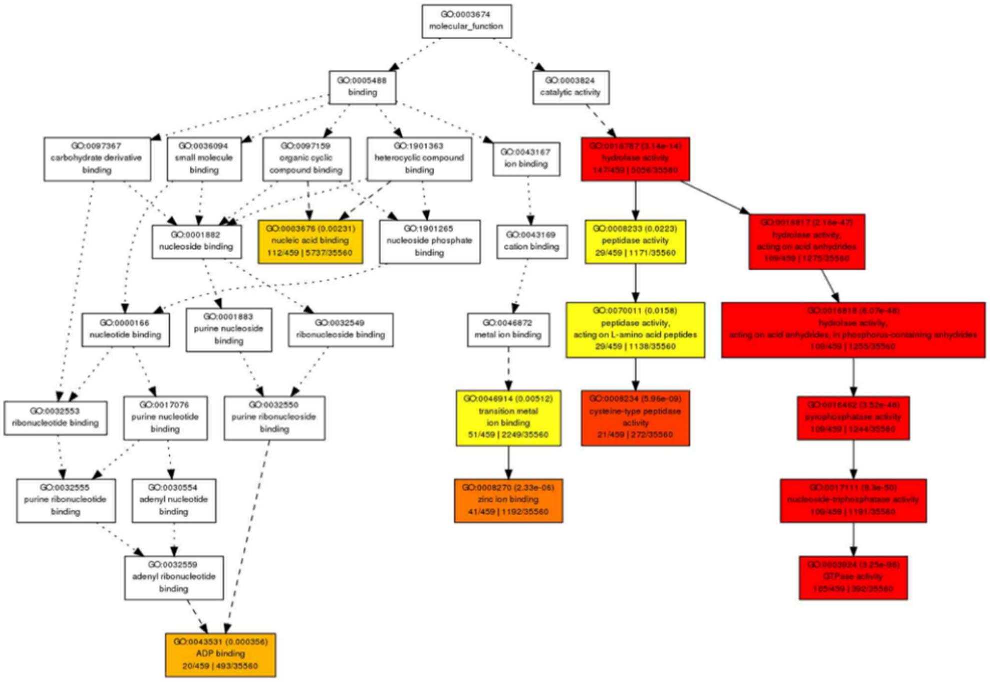

Supplementary Figure 8. GO enrichment of lineage specific genes in broomcorn millet.

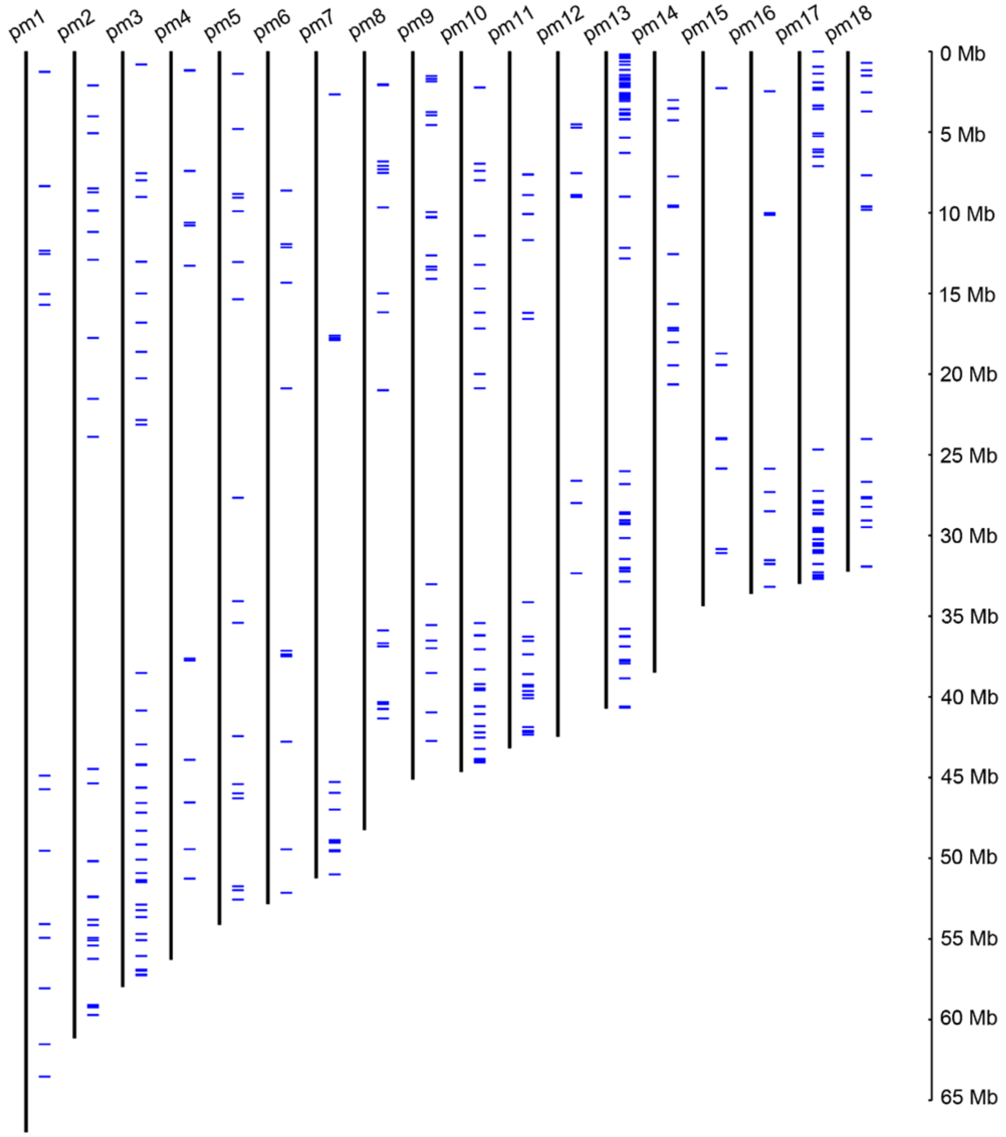

Supplementary Figure 9. Distribution of NB-ARC domain genes (the blue bars) along the 18 pseudomolecules of broomcorn millet.

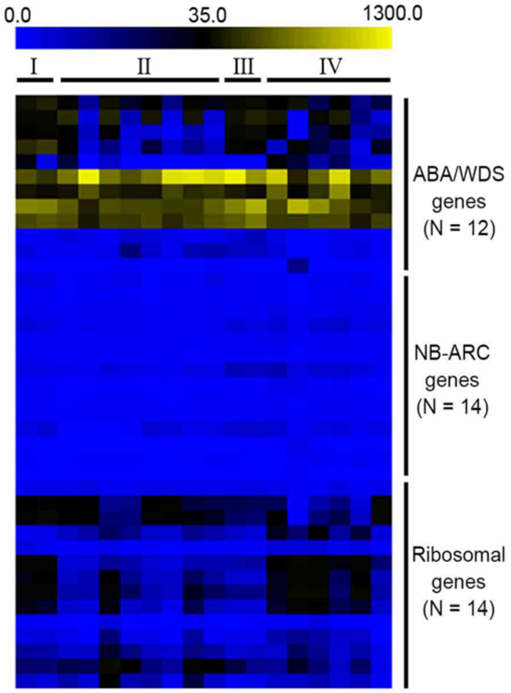

Supplementary Figure 10. Expression profiles of ABA responsive genes in broomcorn millet. We randomly selected some NB-ARC genes and ribosomal genes as controls. The color scale above denotes the FPKM of genes from □. Mixed tissues including leaves, stems, roots, shoots and spikes; □. Leaves with or without salt treatment. □. Leaves with or without drought treatment. □. Samples from 6 developmental stages/tissues, including the firth leaf, root, young spikes, flag leaf, mature spikes, and young leaf.

Supplementary Table 1. Summary of the Illumina, PacBio and BioNano data for the assembly of Longmi4 genome.

Platform	Illumina	PacBio	BioNano
Reads	~68.9 M	~17.5 M	~0.81 M
Data volume	~103.4 Gb	~150.7 Gb	~208.8 Gb
Read length	150 bp	-	-
N50	-	~12.6 Kb	~255.2 Kb
Coverage	~116.4 x	~170.0 x	~235.2 x

Supplementary Table 2. The statistics of genome maps assembled from BioNano data.

BioNano genome maps	Statistics
Number Genome Maps	831
Total Genome Map Length (Mb)	864.321
Mean Genome Map Length (Mb)	1.040
Median Genome Map Length (Mb)	0.769
Genome Map N50 (Mb)	1.445
Total Reference Length (Mb)	848.40
Total Genome Map Length / Reference Length	1.023
Total number of aligned Genome Maps	811 (0.98)
Total Aligned Length (Mb)	802.310
Total Aligned Length / Reference Length	0.949
Total Unique Aligned Length (Mb)	763.945
Total Unique Aligned Length / Reference Length	0.904

Supplementary Table 3. The RNA-seq data and mapping statistics in this study. SE, single end. PE, paired end. *, the mixed tissues including leaves, stems, roots, shoots, and spikes of different growing stages.

SRA accession	Type	Length	Data volume	Mapping efficiency	Genotype	Tissue
ERR2040773	PE	90	1.51 Gb	94.8%	SOHV	Mixed
SRR1697309	PE	101	4.81 Gb	87.7%	HM	Juvenile
SRR1697310	PE	101	4.59 Gb	87.1%	ZY	leave
SRR2179899	SE	51	1.74 Gb	91.0%	NA	
SRR2179900	SE	51	1.70 Gb	91.0%	NA	
SRR2179901	SE	51	1.64 Gb	91.9%	NA	
SRR2179902	SE	51	1.68 Gb	91.9%	NA	
SRR2179903	SE	51	1.66 Gb	91.6%	NA	
SRR2179904	SE	51	1.66 Gb	92.3%	NA	Leaf at 3
SRR2179905	SE	51	1.71 Gb	92.0%	NA	leaves stage
SRR2179906	SE	51	1.71 Gb	92.4%	NA	
SRR2179907	PE	101	4.13 Gb	84.3%	287	
SRR2179908	PE	101	4.72 Gb	83.9%	Laomizi	
SRR2179952	SE	101	1.59 Gb	87.9%	NA	
SRR2179961	SE	101	1.64 Gb	88.6%	NA	
SRR4069168	PE	100	2.86 Gb	94.4%	Yixuan dahongmi	Fifth leaf
SRR4069169	PE	100	2.38 Gb	90.3%	Yixuan dahongmi	Root
SRR4069170	PE	100	2.34 Gb	94.3%	Yixuan dahongmi	Young spikes
SRR4069171	PE	100	2.85 Gb	94.2%	Yixuan dahongmi	Flag leaf
SRR4069172	PE	100	2.22 Gb	93.8%	Yixuan dahongmi	Mature spike
SRR4069173	PE	100	2.76 Gb	94.3%	Yixuan dahongmi	Young leaf
Yue, et al.	PE	101	4.81 Gb	95.9%	Yumi3	Mixed
2016.	PE	101	5.41 Gb	96.4%	Yumi3	tissues*
Generated in this study	PE	100	6.46 Gb	92.9%	Longmi4	Seedlings
Total	-	-	68.6 Gb	91.5%	-	-

Supplementary Table 4. The mapping statistics of *in vivo* Hi-C libraries. Totally, ~622.2 million paired-end reads (100 bp) were generated which covered ~140.2x of Longmi4 genome. Only unique paired alignments (~115.6 million) were used for downstream analysis, and the valid interaction pairs (~64.9 million) were used to build the interaction matrices.

Mapping information	Read pairs	Percentage
Total pairs processed	622,265,931	100%
Unmapped pairs	107,947,306	18.0%
Low quality pairs	0	0%
Unique paired alignments	115,588,452	17.9%
Multiple pairs alignments	76,394,211	11.8%
Pairs with singleton	322,335,962	52.2%
Low quality singleton	0	0%
Unique singleton alignments	0	0%
Multiple singleton alignments	0	0%
Reported pairs	115,588,452	17.9%
Valid interaction pairs	64,928,483	10.4%
Dangling end pairs	11,806,471	1.9%
Re-ligation pairs	3,314,331	0.5%
Self cycle pairs	2,991,643	0.5%
Single-end pairs	0	0%
Dumped pairs	32,547,524	5.2%

Supplementary Table 5. The statistics of pseudomolecules constructed according to the Hi-C interaction matrices. The pseudomolecules were named according to the length. Pm, *Panicum miliaceum*. Si, *Setaria italica*.

Pseudomolecules	Length (bp)	Scaffold number	Gene number	Homolog chromosome in Yugu1
Pm1	69,183,459	41	5,688	Si9
Pm2	61,153,219	29	4,320	Si2
Pm3	57,970,102	19	4,318	Si3
Pm4	56,286,655	17	5,237	Si9
Pm5	54,126,031	24	4,566	Si5
Pm6	52,839,179	23	3,749	Si1
Pm7	51,234,605	30	2,908	Si4
Pm8	48,259,421	18	4,252	Si5
Pm9	45,112,342	40	2,517	Si6
Pm10	44,648,547	28	3,929	Si3
Pm11	43,177,482	15	3,850	Si2
Pm12	42,466,157	15	3,488	Si1
Pm13	40,720,392	29	1,839	Si8
Pm14	38,490,750	17	2,834	Si7
Pm15	34,360,906	20	2,804	Si7
Pm16	33,613,985	36	2,567	Si4
Pm17	32,993,148	17	1,811	Si8
Pm18	32,237,550	26	2,257	Si6
Total	838,873,930	444	62,934	-

Supplementary Table 6. Comparison of gene models between broomcorn millet, foxtail millet, pearl millet, sorghum and maize.

Species	Gene number	Mean transcript length (bp)	Mean CDS length (bp)	Mean intron length (bp)
Broomcorn millet	63,671	~2,883	~1,023	~1,270
Foxtail millet	34,584	~2,073	~1,249	~1,495
Pearl millet	35,791	~2,420	~1,021	~1,416
Sorghum	33,235	~2,111	~1,204	~1,760
Maize	39,498	~3,789	~1,447	~3,510

Supplementary Table 7. The classification of duplicated genes in broomcorn millet and foxtail millet. *Only genes mapped in pseudomolecules were analyzed.

Species	Total genes*	WGD or segmental	Tandem	Proximal	Dispersed	Singleton
Broomcorn millet	62,934	39,769 (~63.2%)	2,712 (~4.3%)	2,063 (~3.3%)	13,142 (~20.9%)	5,248 (~8.3%)
Foxtail millet	34,264	5,805 (~16.9%)	4,356 (~12.7%)	2,166 (~6.2%)	14,570 (~42.5%)	7,367 (~21.5%)

Supplementary Table 8. The identification of syntenic genes between foxtail millet (Si1~Si9) and the two subgenomes of broomcorn millet. Since no biased fractionation of duplicated genes was observed in broomcorn millet, we classified the two homologous chromosomes into two subgenomes according to the chromosome length.

Reference	Total genes	Syntenic genes	Subgenome1	Subgenome2	Both
Si1	3,808	2,415	2,245 (Pm6)	2,299 (Pm12)	2,129
Si2	4,455	2,494	2,296 (Pm2)	2,314 (Pm11)	2,131
Si3	4,096	2,486	2,345 (Pm14, Pm3)	2,300 (Pm15, Pm10)	2,159
Si4	2,925	1,598	1,471 (Pm7)	1,484 (Pm16)	1,360
Si5	4,712	2,870	2,726 (Pm5)	2,713 (Pm8)	2,569
Si6	2,561	1,304	1,202 (Pm9)	1,164 (Pm18)	1,062
Si7	3,359	1,871	1,636 (Pm14, Pm3)	1,664 (Pm15, Pm10)	1,467
Si8	2,535	833	676 (Pm13)	735 (Pm17)	578
Si9	5,813	3,657	3,443 (Pm1)	3,467 (Pm4)	3,258
Other scaffolds	320	81	NA	NA	NA
Total	34,584	19,609	18,040	18,140	16,884

Supplementary Table 9. Transcription factor genes in Longmi4.

TF family	Numbers	TF family	Numbers	TF family	Numbers
AP2	42	G2-like	87	NF-YA	18
ARF	41	GATA	53	NF-YB	29
ARR-B	16	GeBP	23	NF-YC	28
B3	105	GRAS	125	Nin-like	25
BBR-BPC	6	GRF	19	RAV	6
BES1	11	HB-other	17	S1Fa-like	2
bHLH	295	HB-PHD	4	SBP	33
bZIP	171	HD-ZIP	87	SRS	12
C2H2	177	HRT-like	2	TALE	44
C3H	81	HSF	35	TCP	30
CAMTA	11	LBD	69	Trihelix	58
CO-like	24	LFY	2	VOZ	4
CPP	18	LSD	8	Whirly	4
DBB	18	MIKC_MADS	50	WOX	24
Dof	60	M-type_MADS	40	WRKY	164
E2F/DP	12	MYB	237	YABBY	17
EIL	17	MYB_related	129	ZF-HD	29
ERF	293	NAC	243	NF-X1	4
FAR1	114				

Supplementary Table 10. The proportion of repeat elements in broomcorn millet, foxtail millet and pearl millet.

Class	SuperFamilies	Broomcorn millet	Foxtail millet	Pearl millet
Retrotransposons		37.08%	29.57%	55.19%
	Copia	4.38%	5.55%	16.63%
	Gypsy	31.37%	22.04%	37.28%
	LINE	0.98%	1.59%	0.97%
	SINE	0.17%	0.12%	0.13%
DNA transposons		4.85%	10.21%	4.76%
	hAT	0.62%	0.61%	0.36%
	CMC-EnSpm	2.49%	5.16%	2.98%
	MULE-MuDR	0.57%	1.37%	0.64%
	PIF-Harbinger	0.62%	2.28%	0.48%
Helitrons		0.44%	0.63%	0.11%
Simple_repeats		0.87%	0.74%	0.30%
rRNAs		0.03%	0.20%	-
Unclassified		8.23%	5.30%	7.52%
Total		54.09%	46.84%	68.01%

Supplementary Table 11. The statistics of raw contigs.

Type	Contig Length (bp)	Contig number
N50	2,580,906	85
N60	1,927,663	123
N70	1,404,846	174
N80	937,134	247
N90	483,621	370
Longest	19,184,024	1
Total	838,024,289	1,262
Length>=1Kb	838,023,630	1,249
Length>=2kb	838,006,909	1,060

Supplementary Table 12. The statistics of conflicts-resolved contigs and scaffolds.

Type	Scaffold length (bp)	Scaffold number	Contig length (bp)	Contig number
N50	8,243,672	31	2,552,491	87
N60	6,065,463	44	1,864,380	126
N70	4,031,103	61	1,354,575	178
N80	2,951,927	56	875,370	256
N90	1,474,393	127	455,341	387
Longest	22,633,379	1	19,200,716	1
Total	848,394,418	905	838,971,671	1,308
Length >= 1kb	848,393,579	900	838,970,832	1,303
Length >= 5kb	848,256,705	853	838,833,958	1,256

Supplementary Reference

1. Cox, M.P., Peterson, D.A. & Biggs, P.J. SolexaQA: At-a-glance quality assessment of Illumina second-generation sequencing data. *BMC bioinformatics* **11**, 485 (2010).
2. Marçais, G. & Kingsford, C. A fast, lock-free approach for efficient parallel counting of occurrences of k-mers. *Bioinformatics* **27**, 764-770 (2011).
3. Lamichhaney, S. et al. Structural genomic changes underlie alternative reproductive strategies in the ruff (Philomachus pugnax). *Nature Genetics* **48**, 84 (2016).
4. Vurture, G.W. et al. GenomeScope: fast reference-free genome profiling from short reads. *Bioinformatics* **33**, 2202-2204 (2017).
5. Chin, C. et al. Phased diploid genome assembly with single-molecule real-time sequencing. *Nature methods* **13**, 1050-1054 (2016).
6. Chaisson, M.J. & Tesler, G. Mapping single molecule sequencing reads using basic local alignment with successive refinement (BLASR): application and theory. *BMC bioinformatics* **13**, 238 (2012).
7. Li, H. Aligning sequence reads, clone sequences and assembly contigs with BWA-MEM. *arXiv preprint arXiv:1303.3997* (2013).
8. Walker, B.J. et al. Pilon: an integrated tool for comprehensive microbial variant detection and genome assembly improvement. *PloS one* **9**, e112963 (2014).
