# Supplementary material for: Chromosome conformation capture resolved near complete genome assembly of broomcorn millet
Source: Nat Commun. 2019 Jan 25;10:464. doi: 10.1038/s41467-018-07876-6 (PMC6347627; doi:10.1038/s41467-018-07876-6)
Supplement: Supplementary file 3 — Reporting Summary [file 41467_2018_7876_MOESM3_ESM.pdf]

nature research

Corresponding author(s): Jinsheng Lai

Reporting Summary

Nature Research wishes to improve the reproducibility of the work that we publish. This form provides structure for consistency and transparency in reporting. For further information on Nature Research policies, see Authors & Referees and the Editorial Policy Checklist.

Statistical parameters

When statistical analyses are reported, confirm that the following items are present in the relevant location (e.g. figure legend, table legend, main text, or Methods section).

n/a	Confirmed	
☐	☒	The exact sample size (n) for each experimental group/condition, given as a discrete number and unit of measurement
☒	☐	An indication of whether measurements were taken from distinct samples or whether the same sample was measured repeatedly
☒	☐	The statistical test(s) used AND whether they are one- or two-sided<br>*Only common tests should be described solely by name; describe more complex techniques in the Methods section.*
☒	☐	A description of all covariates tested
☒	☐	A description of any assumptions or corrections, such as tests of normality and adjustment for multiple comparisons
☒	☐	A full description of the statistics including central tendency (e.g. means) or other basic estimates (e.g. regression coefficient) AND variation (e.g. standard deviation) or associated estimates of uncertainty (e.g. confidence intervals)
☒	☐	For null hypothesis testing, the test statistic (e.g. F, t, r) with confidence intervals, effect sizes, degrees of freedom and P value noted<br>*Give P values as exact values whenever suitable.*
☒	☐	For Bayesian analysis, information on the choice of priors and Markov chain Monte Carlo settings
☒	☐	For hierarchical and complex designs, identification of the appropriate level for tests and full reporting of outcomes
☒	☐	Estimates of effect sizes (e.g. Cohen's d, Pearson's r), indicating how they were calculated
☒	☐	Clearly defined error bars<br>*State explicitly what error bars represent (e.g. SD, SE, CI)*

Our web collection on statistics for biologists may be useful.

Software and code

Policy information about availability of computer code

Data collection	No software was used during data collection
Data analysis	Falcon (v1.8.7), Blasr (v5.1), Arrow (v2.1.0), Pilon (v1.20), IrysSolve (BioNano Genomics), bowtie2 (v2.0.5), HiC-Pro (v2.7.8), Juicebox (v1.8.8), Lachesis (https://github.com/shendurelab/LACHESIS), Tophat2 (v2.1.1), Cufflinks (2.2.1), Repeatmasker (open-4.0.7), Fgenesh (v.3.1.1), blastall (v2.2.26), InterProScan (v 5.15-54.0), AgriGO (version 2, http://systemsbiology.cau.edu.cn/agriGOv2/), OrthoMCL, MCScanX (http://chibba.pgml.uga.edu/mcscan2/), RepeatModeler (open-1.0.11), RepeatMasker (open-4.0.7), LTRharvest (v1.5.9), MUSCLE (v3.8.31), EMBOSS (v6.6.0), Coge (https://genomevolution.org/coge/)

For manuscripts utilizing custom algorithms or software that are central to the research but not yet described in published literature, software must be made available to editors/reviewers upon request. We strongly encourage code deposition in a community repository (e.g. GitHub). See the Nature Research guidelines for submitting code & software for further information.

Data

Policy information about availability of data

All manuscripts must include a data availability statement. This statement should provide the following information, where applicable:

- Accession codes, unique identifiers, or web links for publicly available datasets
- A list of figures that have associated raw data
- A description of any restrictions on data availability

Genome assembly was deposited into NCBI Genbank with accession ID PPDP00000000. The genome assembly and annotations have also been deposited in the Genome Warehouse in BIG Data Center, Beijing Institute of Genomics (BIG), Chinese Academy of Sciences, under accession number GWHAAEZ00000000 that is publicly accessible at http://bigd.big.ac.cn/gwh. The genome sequence and gene annotations could also be found at Coge (https://genomevolution.org/coge/) with genome ID 50980. The transcriptome data, Illumina reads (Longmi4 resequencing and Hi-C) and PacBio bam files generated in this study were deposited into NCBI SRA with accession number SRP128667.

Field-specific reporting

Please select the best fit for your research. If you are not sure, read the appropriate sections before making your selection.

☒ Life sciences ☐ Behavioural & social sciences ☐ Ecological, evolutionary & environmental sciences

For a reference copy of the document with all sections, see nature.com/authors/policies/ReportingSummary-flat.pdf

Life sciences study design

All studies must disclose on these points even when the disclosure is negative.

Sample size	No predetermination of the sample size was needed. Sufficient fresh seedlings were used to extract DNA for PacBio sequencing.
Data exclusions	No data were excluded for the analysis.
Replication	No replications related to in this study.
Randomization	No randomization was needed in this study.
Blinding	Blinding was not relevant to this study.

Reporting for specific materials, systems and methods

Materials & experimental systems

n/a	Involved in the study
☒	☐ Unique biological materials
☒	☐ Antibodies
☒	☐ Eukaryotic cell lines
☒	☐ Palaeontology
☒	☐ Animals and other organisms
☒	☐ Human research participants

Methods

n/a	Involved in the study
☒	☐ ChIP-seq
☒	☐ Flow cytometry
☒	☐ MRI-based neuroimaging
